# The Roles of Empathy, Proximity, and Identity in Alzheimer’s Disease Stigma

**DOI:** 10.3390/bs16040525

**Published:** 2026-04-01

**Authors:** Adriana Seda, Ellie Mitova, Aubrianna L. Stuckey, Steven L. Berman

**Affiliations:** 1 Department of Psychology, University of Central Florida, Sanford, FL 32773, USAellie.mitova@ucf.edu (E.M.); 2 Department of Counseling, Educational Psychology and Research, College of Education, University of Memphis, Memphis, TN 38125, USA; lstuckey@memphis.edu

**Keywords:** Alzheimer’s Disease, stigma, identity distress, internalization, symbolization, empathy, moral identity, identity

## Abstract

Stigma toward individuals with Alzheimer’s Disease (AD) can have significant social and psychological consequences, yet research on its contributing factors to stigmatizing attitudes remain limited. The current study represents a novel investigation into stigma toward individuals with AD, a population that has been largely overlooked in studies of stigmatization. The present study aimed to examine psychosocial predictors of AD stigma, focusing on empathy, moral identity, identity distress, and proximity, with individuals with AD as a potential protective factor. A sample of undergraduate students (*N* = 419) completed self-report measures assessing these constructs. Empathy was the strongest predictor of AD stigma, negatively related to stigmatizing attitudes and fully mediating the relationship between moral identity internalization and stigma. In contrast, identity distress was linked to higher stigma, both directly and indirectly through its negative association with empathy. Proximity to someone with AD was also associated with lower stigma, suggesting that proximity may promote more positive attitudes. These findings highlight the possible central role of empathy in mitigating stigma and suggest that interventions fostering empathic concern, alongside addressing identity-related distress, may help mitigate negative perceptions of individuals with AD.

## 1. Introduction

### 1.1. Alzheimer’s Disease Perception and Stigma

Defining features of Alzheimer’s Disease (AD) include significant decline from a previous performance in cognitive domains such as executive function, learning and memory, social cognition, attention, language and perceptual motor skills (American Psychiatric Association, 2022). For the general population, memory loss is the most recognized feature of AD, which involves difficulty in learning new information (Weiner et al., 2009). According to the Alzheimer’s Association (2024), an estimated 6.9 million people in the United States are diagnosed with AD, and by 2060, the number is projected to reach 13.8 million.

In recent years, research regarding AD dementia has advanced such that it is currently conceptualized in three different stages. Specifically, the National Institute on Aging—Alzheimer’s Association (NIA-AA) proposed to divide AD into the preclinical, prodromal, and AD dementia stages (Jack et al., 2018). The preclinical stage refers to those who have the presence of the abnormal biomarkers associated with AD but without the cognitive impairment. The prodromal stage (i.e., Mild Cognitive Impairment) has both abnormal biomarkers and cognitive impairment, while the AD dementia stage, the one our society is most familiar with, is defined by severe cognitive impairment (Rosin et al., 2020).

Despite the prevalence of this disease in the country, there have been several studies that have demonstrated that there is a stigma surrounding people with AD (Harper et al., 2019; Nguyen & Li, 2020; Stites et al., 2018; Urbańska et al., 2015). Stigma, as defined by Goffman (1963), is an attitude or behavior that socially discredits an individual and causes others to regard the individual as an undesirable and rejected member of society. Stigma towards people with AD usually involves patronizing, isolating, and discriminatory behaviors (Corner & Bond, 2004; Werner & Giveon, 2008).

There is no cure for Alzheimer’s Disease currently, and much remains unknown regarding what causes it. Thus, clinical research is still very prevalent on this specific neurodegenerative disease in order to further our understanding of it. Specifically, there is a stigma that people with AD can be dangerous or that they need to be avoided (i.e., social distance; Nguyen & Li, 2020). These behaviors, in turn, can have a negative effect on the AD community, perhaps explaining why people with AD reported feeling incompetent, unable to participate in social events, having a lower self-esteem, feeling frustrated, depressed, ashamed, embarrassed, etc. (Nguyen & Li, 2020; Urbańska et al., 2015). The stigma that surrounds AD might prevent people from participating in AD clinical trials or they might attempt to conceal any symptoms they may be experiencing to avoid getting a diagnosis (Rewerska-Juśko & Rejdak, 2020; Rosin et al., 2020; Urbańska et al., 2015). According to the framework proposed by the NIA-AA (Jack et al., 2018), some people might be diagnosed with AD without even having the symptoms of cognitive impairment that everyone is familiar with (i.e., preclinical AD); hence, some people might be hesitant to participate in the clinical trial for earlier stages of AD because they could get an AD diagnosis without having the symptoms yet (Rosin et al., 2020).

The stigma experienced by those with AD is brought upon from the multiple factors and misconceptions that surround AD. Several research studies have explored AD perception in different ethnic groups, showing that individuals that identified as Hispanic/Latinos and Asians tended to consider AD as an infectious entity that can be cured (Ayalon & Areán, 2004; Blay & Peluso, 2010; Rewerska-Juśko & Rejdak, 2020), Israeli Arabs tended to believe the disease is in the control of God (Cohen et al., 2009; Rewerska-Juśko & Rejdak, 2020), and Indigenous Australians tended to believe that having dementia is a luxury from living a lengthy life (Cipriani & Borin, 2014; Nguyen & Li, 2020). All these factors together add to the belief that a diagnosis of AD is a “death sentence” regardless of what stage of the disease the patient is in (Rosin et al., 2020; Urbańska et al., 2015). Although, there is another group of people on the other end of the spectrum that consider dementia to be a normal part of aging and not a neurodegenerative disease or a “death sentence” (Alzheimer’s Disease International, 2019; Rewerska-Juśko & Rejdak, 2020).

Furthermore, Low and Purwaningrum (2020) systematically reviewed papers across the Scopus, PubMed, PsychInfo, and Embase databases that studied the depiction of AD in popular culture (i.e., social media, literature, and film). They reviewed 60 papers that covered a wide array of media classifications such as television, newspapers, and literature. One of their findings was that the most common type of dementia represented was AD, and in most cases, AD and dementia were used interchangeably. Books and films typically had a plot that revolved around the progressive decline or eventual death of the person with dementia. The review determined that, across the different forms of media platforms, the majority depicted dementia negatively. The representation of an individual with AD in the media included loss of memory, identity, unpredictable behavior, and underlying suffering. This portrayal of AD in the media dehumanizes people diagnosed with it and evokes social distance from others.

Oscar et al. (2017) also analyzed AD representation on social media. They developed software that analyzed 31,150 tweets related to AD on Twitter. This software stratified the tweets into six categories: informative, joke (e.g., if Returra had memory loss and forgot he had class right now, that would be great), metaphorical, organization, personal experience, and ridicule (e.g., waiting until your grandparents become senile so you can trick them into giving you their money). Their findings reflected that a substantial amount of the tweets were classified under the category of ridicule, exemplifying the negative views of individuals with AD.

Johnson et al. (2015) explored which aspects of AD evoke more stigma: the label of AD or the observable symptoms. They claimed that understanding where the stigma stemmed from would lay the groundwork for the consequences that could arise from explaining AD as a spectrum that includes a preclinical stage [i.e., presence of abnormal biomarkers with no cognitive symptoms]. The participants (*N* = 789) took a survey that randomized them to read one of nine vignettes of a man displaying symptoms of mild AD and whether the symptoms would worsen, improve, or stay the same. The results revealed that disease prognosis was the most significant contributor to social distance. This suggests that the negative reactions towards people with AD stem not from the label of the disease itself but rather from the prognosis that is expected from the disease.

Previous research suggests that younger individuals exhibit a greater stigma toward individuals with AD compared to their older counterparts. For example, younger individuals have been shown to report higher levels of stigmatizing attitudes toward people with AD and are more likely to express feelings of pity toward those diagnosed with the condition (Johnson et al., 2015). Additionally, when responding to a questionnaire assessing hypothetical reactions to receiving an AD diagnosis, younger participants reported greater anticipated shame and loss of self-esteem (Piver et al., 2012). In contrast, older participants were less likely to stigmatize those with AD across different studies (Harper et al., 2019; Nguyen & Li, 2020; Rewerska-Juśko & Rejdak, 2020; Stites et al., 2018), and this could be because older individuals have a greater perceived personal risk of developing AD and/or their understanding of the disease through their experiences with closer relatives or friends (Stites et al., 2018). Since older individuals tend to express less stigma towards those diagnosed with AD, positive media visibility should be targeted towards younger adults, i.e., the social group that expresses the most stigma. As suggested by O’Connor and McFadden (2012), it will be hard to combat the ageism associated with AD without an effort to change the narrative that surrounds those with AD (e.g., AD is a death sentence).

Based on the current literature, there is substantial stigma towards individuals with AD, and this study aimed to further explore alternative factors and their role in the stigmatization of AD dementia, with a focus on younger adults who have been shown to report higher stigmatizing attitudes.

### 1.2. Alzheimer’s Disease and Proximity

Proximity to an individual with AD might mitigate stigma due to the individual’s close relationship with the person diagnosed with AD. The current literature has explored the relationship between AD stigma and proximity, primarily amongst caregivers, suggesting that knowing someone with AD can decrease the level of stigma towards them (Werner & Davidson, 2004). This association may be due to the individual being more knowledgeable about AD, which in turn can lead to more positive attitudes about the disease (Teichmann et al., 2022). Nonetheless, there has been research contradicting the idea that close proximity to an individual with AD lessens stigmatizing behaviors. Stites et al. (2021) found that caregivers are more capable of stigmatizing people with AD compared to non-caregivers despite close contact with them. This finding was also consistent with a study amongst Japanese citizens, which found that close social proximity to someone with dementia can increase stigma, but the effect can be mitigated by being knowledgeable about the disease (Ito & Tsuda, 2025). These results beg the question as to whether the proximity factor is mitigating the stigma associated with AD or if it is because those who are more intimately acquainted with individuals with AD are more empathetic towards them and, therefore, they are less likely to stigmatize them. Werner (2005) conducted face-to-face interviews with Jewish Israelis (*N* = 206) to examine social determinants of social distance towards individuals with AD. They found that proximity with someone with AD reduces the incidence of social discrimination through the mediating effect of prosocial emotions such as sympathy, desire to help, concern, consternation, and compassion. Nonetheless, the effect of proximity to someone with AD on stigma remains a contradiction.

A possible explanation could be that those that are associated with someone with AD might be the recipients of stigma themselves. This phenomenon is called “spillover stigma”, and it occurs when the stigma towards a specific population extends to the caregivers and those who are associated with them (Goffman, 1963; Stites et al., 2021). Goffman (1963) suggested that people who have any sort of relationship with a stigmatized individual can be seen as “one” with them, therefore affecting both parties. This dilemma might be dependent on how impaired the individual with AD is and how noticeable this is to the general population. However, proximity can affect different groups of people differently. For example, family members and/or caregivers might not necessarily feel personally affected by spillover stigma (MacRae, 1999). Neighbors of a small community are also less likely to stigmatize an individual that they know intimately, whereas close friends might put social distance between themselves and the person with AD (MacRae, 1999).

Due to the inconsistent findings surrounding the association between proximity and social distance (i.e., a stigmatizing behavior), this study aims to explore whether proximity plays a role in reducing stigma towards individuals with AD.

### 1.3. Alzheimer’s Disease and Empathy

Empathy is the ability to understand another person’s feelings and experiences without losing awareness of one’s own feelings; it is a sense of similarity between another person and oneself (Decety & Jackson, 2004). Empathy may be a motivator that induces people to engage in socially moral behaviors due to the moral implications that underlie it (e.g., sending money to help famine victims halfway around the world; Batson et al., 2015; Decety & Cowell, 2015).

Although the association between empathy and AD has been scarcely studied, Batson et al. (2015) found that perspective-taking can evoke empathy for a member within certain stigmatized groups. Empathetic responses, in turn, have been shown to foster more favorable attitudes towards highly stigmatized groups such as people with AIDS, homeless individuals, and convicted murderers (Batson et al., 1997). Notably, this effect occurred irrespective of perceptions of personal responsibility for their situation. Additionally, in a survey where respondents were primed with an intervention that encouraged positive empathetic concern, they were less likely to endorse social distance (i.e., a behavioral response of stigma) towards people with a substance use disorder, which is another stigmatized group (Clinton & Pollini, 2021). Hamed et al. (2025) found a negative association between stigmatizing attitudes and empathy amongst psychiatric nurses towards patients with mental illnesses, as higher stigmatizing attitudes were linked to lower levels of empathy. Overall, while existing research demonstrates a consistent link between empathy and reduced stigma across various stigmatized groups, very little is known about how empathy relates specifically to stigma toward individuals with AD dementia.

Empathy has also been identified as a commonly evoked emotional response among younger individuals when they consider older adults, particularly those with AD (O’Connor & McFadden, 2012). It is often characterized as a positive emotion that may reduce discriminatory behaviors toward individuals diagnosed with dementia (Nguyen & Li, 2020). Evidence further suggests that having a close relative with dementia can lessen stigmatizing attitudes, likely due to the prosocial emotions elicited though close personal contact (Werner, 2005; Werner & Davidson, 2004). Similarly, a study of British adolescents found that higher empathy and closer contact with a person living with AD were associated with lower levels of dementia-related stigma (Hassan et al., 2024). However, despite people with AD eliciting comparatively high empathy ratings, they are often perceived as less competent than individuals with other conditions, such as arthritis, making it unclear whether empathy alone is sufficient to reduce discrimination and stigma towards those with AD (O’Connor & McFadden, 2012).

This study examines whether higher levels of empathy are associated with reduced stigma toward individuals with AD, thereby attempting to replicate findings observed in research on other stigmatized groups (Batson et al., 1997; Clinton & Pollini, 2021; Hamed et al., 2025).

### 1.4. Alzheimer’s Disease and Moral Identity

Stigma has been described as a moral issue because it disrupts the everyday interpersonal experiences of both those who stigmatize and those who are stigmatized (Yang et al., 2007). This is due to the fact that moral standing depends on fulfilling social obligations and norms, which stigmatized individuals are often seen as unable to do (Kleinman & Hall-Clifford, 2009). Moral identity has been argued to be the underlying construct that links empathy and prosocial behavior (Peng et al., 2024). Moral identity is defined as identifying oneself with a set of moral traits such as being fair, generous, and/or kind (Aquino & Reed, 2002). Aquino and Reed II suggest that there are two dimensions of moral identity: internalization and symbolization. Internalization refers to how important those moral traits are to oneself, and symbolization is the degree to which those moral traits are expressed through actions/behaviors (Aquino & Reed, 2002).

Moral identity has also been found to moderate the process from moral emotions to prosocial behavior (Ding et al., 2018). College students who were more empathetic also reported a stronger sense of moral identity, which in turn promoted greater prosocial behaviors (Peng et al., 2024). Symbolization can also increase prosocial behaviors but only in individuals whose level of internalization is low (Winterich et al., 2013). Those who are high in internalization are more likely to engage in prosocial behaviors regardless of whether they get recognized for it, whereas those who are high in symbolization might engage in prosocial behavior but only if it is contingent on getting recognized for it (Winterich et al., 2013).

Symbolization has been described as the external or behavioral side of moral identity (Aquino & Reed, 2002); thus, its relationship to prosocial behaviors has been explored in the literature (Ding et al., 2018; Gotowiec & van Mastrigt, 2019; Reynolds & Ceranic, 2007; Winterich et al., 2013). Unlike internalization, symbolization has been associated with behaviors such as volunteering (Aquino & Reed, 2002) or charitable donations (Reynolds & Ceranic, 2007); in other words, actions that can be perceived by others. However, Gotowiec and van Mastrigt (2019) found that high symbolization was associated with a dominant effect on both public and private behaviors regardless of the level of internalization. Internalization does have more robust moderating effects because of how personal and reflective it can be as compared to symbolization (Reynolds & Ceranic, 2007), hence the reason it is more commonly discussed. Nonetheless, these findings reinforce how important it is to include both dimensions of moral identity to get an accurate representation of its role in different situations (Gotowiec & van Mastrigt, 2019).

The effect of moral identity on stigma towards people with AD has not been explored yet. However, considering internalization is associated with a lower endorsement of ageism (Dzumba et al., 2018), higher engagement in prosocial behaviors (Ding et al., 2018; Winterich et al., 2013), and empathy (Peng et al., 2024), which has been linked to lower levels of stigma (Batson et al., 1997; Clinton & Pollini, 2021; Hamed et al., 2025; Hassan et al., 2024; Nguyen & Li, 2020; O’Connor & McFadden, 2012), it is hypothesized that higher levels of internalization will be associated with lower levels of AD stigma. Thus, in this study, the focus was on whether having a higher sense of internal moral identity (i.e., internalization) might be associated with empathy and if this effect can mitigate stigmatizing behaviors towards those with AD.

### 1.5. Alzheimer’s Disease and Identity Distress

Another psychosocial factor that may influence AD stigma is identity distress, a form of maladaptive identity development. Identity distress is the result of the negative feelings that arise from the inability to resolve identity issues across different domains (e.g., long-term goals, career choices, and religious identification; Berman, 2020).

Previous studies on identity crisis and stigma have been focused on the viewpoint of the stigmatized individual. Individuals that consider themselves as part of a stigmatized community (e.g., HIV/AIDS, mental illness, and epilepsy) are more likely to experience psychological distress, specifically depression and anxiety (Quinn & Chaudoir, 2009). However, there are no previous studies on the effects of identity distress on the individual that is actively stigmatizing other individuals.

From an outsider’s point-of-view, witnessing someone in need can evoke feelings of empathy and/or personal distress (Carrera et al., 2012). Empathy is more altruistic in the sense that it motivates a need to reduce the other person’s discomfort, whereas personal distress is more egoistic, and the focus is on reducing one’s own aversive arousal (Batson et al., 1983). People that score higher on personal distress are less likely to engage in helping behavior, because the focus is on alleviating one’s own discomfort, not others (Carrera et al., 2012). Although personal distress and identity distress are two different concepts, both do reflect a state of distress that can negatively impair moral behavior. Stigma may evoke aversion responses, similar to personal distress, and it can result in avoidant behavior (Pryor et al., 2009). Considering that social distance is a common reaction towards people with AD (Nguyen & Li, 2020), this study assesses whether those experiencing high identity distress have a higher stigma toward AD, a concept that has not been addressed in the current literature.

### 1.6. Rationale

The broader stigma literature has shown that various psychological and social factors may shape the degree to which individuals hold negative perceptions of people with conditions that evoke fear, discomfort, or misunderstanding. However, research specifically examining AD stigma from the standpoint of the stigmatizer, rather than the individual experiencing the stigma, remains notably limited. While the consequences of perceived stigma among individuals with AD are well documented, including increased feelings of incompetence, depression, and reduced self-esteem, far less is known about the factors that influence the development of stigmatizing attitudes in the general public. The present study seeks to address this gap by examining several psychosocial variables that may contribute to the formation or reduction in stigma toward people with AD. This study focuses on proximity to someone with AD, empathy, moral identity, and identity distress. In this study, moral identity is composed of both internalization and symbolization. Moral identity and identity distress are introduced as novel variables in this context due to their conceptual links to prosocial emotional processes, including empathy, and their potential to shape how individuals perceive and respond to those with cognitive decline. Prior research suggests that proximity to someone with AD can be associated with either more positive attitudes or, conversely, heightened stigma, indicating that its effects may be complex and context-dependent. By integrating established and emerging psychosocial factors, this study aims to provide a more comprehensive understanding of what contributes to stigma toward individuals with AD and to extend current theoretical models of stigma to this population.

Specifically, this study tested the following hypotheses:

1. Empathy, moral identity internalization, and moral identity symbolization will be negatively associated with AD stigma, whereas identity distress will be positively associated with AD stigma.
2. Individuals who personally know someone with AD will report lower levels of AD stigma than those who do not know someone.
3. Empathy will mediate the relationship between both dimensions of moral identity (i.e., internalization and symbolization) and AD stigma, such that higher internalization and symbolization will predict greater empathy, which in turn will predict lower AD stigma.
4. Empathy will mediate the relationship between identity distress and AD stigma, such that higher identity distress will predict lower empathy, which in turn will predict higher levels of AD stigma.

## 2. Materials and Methods

### 2.1. Participants

Participants (*N* = 419) were undergraduate students attending a large metropolitan university in the southeastern region of the United States. Ages ranged from 18 to 62 years old (*M* = 20.46, *SD* = 4.99). Most participants identified as female (62.1%) or male (36.0%), with smaller proportions identifying as non-binary (0.9%), transgender (0.7%), or another gender identity (0.2%). Of the sample, most were freshmen (32.9%), 28.7% were sophomores, 24.0% were juniors, 13.6% were seniors, and 0.6% were “other”. The sample was ethnically diverse, with 45.9% identifying as White, 25.6% as Hispanic or Latino/a, 10.1% as Asian or Pacific Islander, 9.2% as Black, and 9.2% as Mixed or another ethnic background.

### 2.2. Measures

A demographic questionnaire was used to document participants’ age, gender identity, ethnicity, and grade level.

A proximity demographic questionnaire was used to determine whether participants knew an individual diagnosed with Alzheimer’s Disease and, if so, how well they knew that individual on a 5-point scale ranging from 1 (not well at all) to 5 (extremely well).

The Attribution Questionnaire—Short Form was used to assess stigma and discrimination toward individuals with Alzheimer’s Disease and was adapted from its original focus on schizophrenia (Corrigan et al., 2003). The original prompt described a man with schizophrenia, which was changed to a man with Alzheimer’s Disease. Participants were asked to rate, on a 7-point scale ranging from 1 (strongly disagree) to 7 (strongly agree), 27 items regarding their attitudes and behavioral intentions toward the individual. In the present study, Cronbach’s alpha was 0.79 for the total scale. Sample items include “I would be willing to talk to Harry about his problems” and “Harry would terrify me”.

The Toronto Empathy Questionnaire was used to assess participants’ levels of empathy (Spreng et al., 2009). The scale consists of 16 items that measure general emotional empathy. Participants were asked to rate, on a 5-point scale ranging from 1 (never) to 5 (always), 16 items regarding their emotional responses to others. In the present study, Cronbach’s alpha was 0.85 for the total scale. Sample items include “I get a strong urge to help when I see someone who is upset” and “I find it silly for people to cry out of happiness”.

The Self-Importance of Moral Identity Measure was used to assess participants’ moral identity (Aquino & Reed, 2002). The scale consists of 10 items measuring two subscales: internalization and symbolization. Before completing the items, participants were asked to imagine a person with nine moral traits (e.g., caring, fair, and honest). Participants were then asked to rate, on a 5-point scale ranging from 1 (strongly disagree) to 5 (strongly agree), 10 items regarding how central these traits were to their identity. In the present study, Cronbach’s alpha was 0.75 for internalization and 0.62 for symbolization. Sample items for internalization include “It would make me feel good to be a person who has these characteristics” and, for symbolization, include “I strongly desire to have these characteristics”.

The Identity Distress Scale was used to assess distress related to identity development across multiple domains (Berman et al., 2004). The scale consists of 7 items that assess concerns in areas such as religion, sexual orientation, life goals, career choices, personal values, group affiliations, and friendships. Participants were asked to rate, on a 5-point scale ranging from 1 (none at all) to 5 (very severely), 7 items regarding how distressed they felt about their identity. In the present study, Cronbach’s alpha was 0.80 for the total scale. Sample items include “Career choice? (e.g., deciding on a trade or profession, etc.)” and “Values or beliefs? (e.g., feeling confused about what is right or wrong, etc.)”.

### 2.3. Procedure

This study was reviewed and approved by the authors’ Institutional Review Board (IRB). Participants were recruited through the Psychology Department’s research participation system, SONA. Students were able to browse a list of available studies and voluntarily select the one(s) in which they wanted to participate for course credit. Upon selecting this study, participants were redirected to an “Explanation of Research” page, where they were given the option to participate or decline. Those who agreed to participate were directed to the study. All survey materials were administered anonymously and online using Qualtrics, a secure, web-based survey platform widely used for academic research. Qualtrics allows researchers to design complex survey flows and collect responses in real time. Its user-friendly interface enabled participants to complete the survey from any electronic device at their convenience.

## 3. Results

### 3.1. Preliminary Results

A series of statistical analyses were conducted to assess whether any of the study variables had any significant differences among demographic categories. A Pearson correlation matrix showed that age was positively associated with empathy (*r* = 0.13, *p* = 0.009) and negatively associated with identity distress (*r* = −0.12, *p* = 0.014). Age was not correlated to any other variables. See Table 1 for correlations among the study variables.

An independent samples *t*-test was conducted to assess potential gender differences among the study variables. Females scored lower than males on AD stigma (*t*_(415)_ = 1.92, *p* = 0.028) and on AD proximity (*t*_(415)_ = 2.14, *p* = 0.017). Conversely, females scored higher than males in empathy (*t*_(413)_ = −5.05, *p* < 0.001), internalization (*t*_(415)_ = −4.86, *p* < 0.001), and symbolization (*t*_(414)_ = −5.34, *p* < 0.001). No significant gender differences were observed for identity distress.

A One-Way Analysis of Variance (ANOVA) was used to determine if there were any ethnicity or grade differences among the study variables. There were no significant differences in ethnicity; however, there were significant grade differences for AD stigma (*F*_(3,418)_ = 3.34, *p* = 0.019) and empathy (*F*_(3,416)_ = 2.88, *p* = 0.036). A Scheffe post hoc comparison revealed that, for AD stigma, freshman scored significantly higher than seniors (*p* = 0.032). Although there was an overall grade difference for empathy, post hoc analyses did not reveal any significant differences between each grade level.

### 3.2. Main Analysis

#### 3.2.1. Hypothesis 1

A multiple linear regression was conducted to examine whether proximity, internalization, symbolization, average distress rating, and empathy predicted AD stigma. The overall model indicated good model fit (*F*_(5,416)_ = 22.38; *p* < 0.001; *R*^2^ = 0.21; and *R*^2^ adj. = 0.20), with significant predictors including proximity (*β* = 0.16; *t* = 3.68; and *p* < 0.001), symbolization (*β* = 0.15; *t* = 3.35; and *p* < 0.001), and identity distress (*β* = 0.12; *t* = 2.72; and *p* = 0.007), whereas empathy was a significant negative predictor of AD stigma (*β* = −0.40; *t* = −6.98; and *p* < 0.001). Internalization did not significantly predict AD stigma. In this model, empathy was the strongest predictor of AD stigma, showing a robust negative association, whereas the effects of the other predictors are comparatively smaller.

#### 3.2.2. Hypothesis 2

To assess whether individuals who know someone with AD would report lower levels of AD stigma, an independent samples *t*-test was conducted. Individuals who knew someone with AD did report significantly lower levels of AD stigma compared to those who did not (*t*_(368)_ = −3.20; *p* < 0.001; and *d* = 0.63).

#### 3.2.3. Hypothesis 3

To examine whether empathy mediated the relationship between moral identity and AD stigma, consistent with the procedure outlined by Baron and Kenny (1986), a series of stepwise multiple regression analyses were conducted separately for each moral identity dimension. The first set of regression analyses examined whether empathy mediated the relationship between moral identity internalization and AD stigma. In the first regression, internalization was entered as the predictor of empathy. The model demonstrated good fit (*F*_(1,421)_ = 258.79; *p* < 0.001; *R*^2^ = 0.38; and *R*^2^ adj. = 0.38), and internalization was a significant positive predictor of empathy (*β* = 0.62; *t* = 16.09; and *p* < 0.001). Given these results, a stepwise regression was conducted to assess mediation. In the first step, internalization was entered as the sole predictor of AD stigma, and empathy was subsequently added in a second model to assess its potential mediating effect. The first model demonstrated good overall fit (*F*_(1,421)_ = 30.55; *p* < 0.001; *R*^2^ = 0.07; and *R*^2^ adj. = 0.07), with internalization significantly predicting AD stigma (*β* = −0.27; *t* = −6.67; and *p* < 0.001), indicating that higher internalization was associated with lower AD stigma. In the second model, both internalization and empathy were entered as predictors of AD stigma. The second model demonstrated good fit (*F*_(2,420)_ = 39.11; *p* < 0.001; *R*^2^ = 0.16; and *R*^2^ adj. = 0.15), indicating that the combination of internalization and empathy accounted for a substantial proportion of variance in AD stigma. In this model, empathy remained a significant negative predictor of AD stigma (*β* = −0.38; *t* = −6.67; and *p* < 0.001), whereas the effect of internalization was no longer significant. These results are consistent with full mediation, suggesting that internalization is associated with higher empathy, which in turn predicts lower AD stigma.

To determine whether this mediational pattern extended to the symbolization dimension of moral identity, a second set of regression analyses was conducted. In the first regression, symbolization was entered as a predictor of empathy. The regression model was significant (*F*_(1,420)_ = 37.21; *p* < 0.001; *R*^2^ = 0.08; and *R*^2^ adj. = 0.08), with symbolization emerging as a significant positive predictor of empathy (*β* = 0.29; *t* = 6.10; and *p* < 0.001). Symbolization was then entered as a predictor of AD stigma. The regression model was not significant (*F*_(1,422)_ = 0.156; *p* = 0.69; and *R*^2^ < 0.001), indicating that symbolization did not significantly predict AD stigma (*β* = 0.019; *t* = 0.40; and *p* = 0.693). Given the absence of a significant association in this step, no further mediation analyses were conducted for symbolization.

#### 3.2.4. Hypothesis 4

To examine whether empathy mediated the relationship between identity distress and AD stigma, a series of linear regressions were conducted, first testing the association between identity distress and empathy, followed by an assessment of the mediation effect. A regression was run to see whether identity distress was associated with empathy. This model represented good overall fit (*F*_(1,367)_ = 8.09; *p* = 0.005; *R*^2^ = 0.02; and *R*^2^ adj. = 0.02), with identity distress emerging as a significant negative predictor of empathy (*β* = −0.15; *t* = −2.84; and *p* = 0.005). Given these results, a stepwise regression was conducted to assess mediation. In the first step, identity distress was entered as the sole predictor of AD stigma, and empathy was subsequently added in a second model to assess its potential mediating effect. The first model demonstrated good overall fit (*F*_(1,367)_ = 9.33; *p* = 0.002; *R*^2^ = 0.03; and *R*^2^ adj. = 0.02), with identity distress significantly predicting AD stigma (*β* = 0.16; *t* = 3.05; and *p* = 0.002). In the second model, both identity distress and empathy were entered as predictors of AD stigma. The second model demonstrated a slightly better fit than the first model (*F*_(2,366)_ = 39.80; *p* < 0.001; *R*^2^ = 0.18; and *R*^2^ adj. = 0.17), suggesting that the combination of identity distress and empathy accounted for a large proportion of variance in AD stigma. In this model, empathy remained a significant predictor of AD stigma (*β* = −0.40; *t* = −8.28; and *p* < 0.001), as did identity distress (*β* = 0.10; *t* = 2.07; and *p* = 0.039), although the effect of identity distress decreased in significance once empathy was controlled. These results are consistent with a pattern of partial mediation, indicating that identity distress contributed to AD stigma both directly and indirectly through its association with empathy.

## 4. Discussion

The aim of this paper was to establish which psychosocial factors are associated with stigma towards individuals with Alzheimer’s Disease, specifically among undergraduate college students. Relatively older participants reported higher levels of empathy and lower levels of identity distress compared to their younger counterparts. This pattern is consistent with previous research showing that older adults tend to exhibit greater empathy and, consequently, lower levels of stigma toward individuals with AD (Harper et al., 2019; Nguyen & Li, 2020; Rewerska-Juśko & Rejdak, 2020; Stites et al., 2018).

Empathy was found to be the significant predictor for Alzheimer’s Disease stigma, surpassing the influence of both identity distress and moral identity. Our analyses further indicated that individuals with higher levels of empathy, as well as those with greater moral identity internalization, tended to report lower levels of AD stigma. Notably, empathy fully mediated the relationship between internalization and AD stigma, consistent with the idea that the protective effect of internalization on stigmatizing attitudes operates through empathic processes. In regard to symbolization, it was not a significant predictor for AD stigma. Interestingly, those who scored higher in symbolization did also report being more empathetic; however, this did not translate into less AD stigma. Because this study focused on general stigma rather than stigmatized behaviors (e.g., social distance), the significance of symbolization could have been underscored by internalization, which is more representative of internal beliefs rather than actions. These findings provide a novel perspective on the interplay between empathy and moral identity in shaping attitudes towards those with AD. It also highlights the possible importance of targeting empathy in interventions aimed at reducing stigma, as it may serve as a key mechanism through which internalized moral values translate into more positive prosocial attitudes.

Similarly, identity distress was found to be related to AD stigma, both directly and indirectly, through its association with empathy. Specifically, higher levels of identity distress were linked to lower empathy, which in turn was associated with greater AD stigma. This finding highlights the important role identity distress might play in shaping stigmatizing attitudes, consistent with the idea that individuals experiencing greater identity-related challenges may be less able to engage in empathic concern for others. As a result, reduced empathy stemming from heightened identity distress may increase the likelihood of stigmatizing behaviors. These findings are consistent with other studies that have found that higher empathetic concern does diminish stigma towards those with AD and other stigmatized groups (Batson et al., 1997; Clinton & Pollini, 2021; Hamed et al., 2025; Hassan et al., 2024). Nonetheless, it was still debated whether empathy could reduce stigma towards those with AD specifically (O’Connor & McFadden, 2012). Our findings support the notion that empathy might reduce AD stigma and serve as a mediating factor for other variables that contribute to lower levels of stigma as well. The interventions that have been implemented before to reduce identity distress have been focused on teaching others how to think critically regarding personal dilemmas, as well as transformative pedagogy (Berman, 2020). Future interventions should also consider the important role of increasing empathetic concern for those experiencing identity distress in order to reduce their stigmatizing behaviors towards other groups as well as those with AD.

Internalization (Peng et al., 2024) and proximity (Hassan et al., 2024; Werner & Davidson, 2004; Werner, 2005) play a significant role in predicting stigma by influencing attitudes both directly and indirectly through their relationships with empathy (Batson et al., 1997; Clinton & Pollini, 2021; Hamed et al., 2025; Hassan et al., 2024; Nguyen & Li, 2020; O’Connor & McFadden, 2012). In the present study, higher internalization was associated with lower AD stigma through its relationship with empathy, suggesting that individuals with a stronger sense of moral identity may be more capable of empathetic concern, which, in turn, reduces stigmatizing behaviors. Internalization, as a key component of moral identity, has been linked to empathy due to the shared moral foundations that underlie both constructs (Decety & Cowell, 2015). Thus, our findings are consistent with prior studies that have found that moral identity is associated with prosocial behaviors (Ding et al., 2018; Winterich et al., 2013).

On the other hand, identity distress exhibited the opposite pattern: higher levels of identity distress were associated with greater AD stigma. Prior research has established that forms of personal distress can contribute to stigmatizing behaviors (Pryor et al., 2009) and that identity distress can produce negative outcomes not only for members of stigmatized groups but also for those outside of these groups (Quinn & Chaudoir, 2009). In this study, examining identity distress in the context of AD stigma was particularly important, given that personal distress, a different construct that also reflects a state of distress, has been suggested to interact with empathy (Carrera et al., 2012). Our findings highlight how self-related psychological challenges may undermine empathic concern, which can increase stigmatizing attitudes towards individuals with AD.

Furthermore, the role of proximity to someone with AD in shaping stigma has produced mixed findings in the previous literature. Research on caregivers has suggested that closeness to an individual with AD does not necessarily reduce stigma, potentially due to the stigma experienced vicariously through association (Goffman, 1963; Ito & Tsuda, 2025; Stites et al., 2021). However, the current study found that being in close proximity to someone with AD was associated with lower levels of stigma, aligning with prior findings from Werner (2005). It is worth noting that this finding could be due to our population of undergraduate students and not specifically caregivers of individuals with AD, although we cannot dismiss the possibility of some of the respondents to be caregivers themselves to someone with AD. The results are limited to people that are in proximity to someone with AD, and this was associated with lower stigma.

Overall, these findings highlight the complex interplay between empathy, moral identity, identity distress, and proximity in shaping attitudes toward individuals with AD. Internalization appears to promote empathy and reduce stigma, whereas identity distress may hinder empathic responses and increase stigma. Proximity, in contrast, demonstrates potential as a protective factor, emphasizing the role of personal experience and proximity in mitigating negative perceptions.

While this study provides valuable insight into factors predicting Alzheimer’s Disease stigma, several limitations should be considered when interpreting the findings of this study. First, the cross-sectional design precludes any conclusions about causality, meaning that while associations between empathy, internalization, identity distress, proximity, and AD stigma were consistent with theories of causation, these causal relationships cannot be definitively established. Longitudinal studies would help to address this limitation by establishing developmental timelines (which precedes which). Second, the study relied on self-report measures, which may be influenced by social desirability bias, particularly given the sensitive nature of stigma and moral identity. Collateral reports from significant others and/or clinical interviews might be able to instill greater confidence in the validity of responses. Third, the sample consisted primarily of college undergraduate students, which is particularly useful given their likelihood to be in a crucial stage of identity development; however, it may limit the generalizability of the findings to other age groups or populations, such as older adults or professional caregivers of individuals with AD. Future studies should explore these relationships across various age ranges and developmental stages. Fourth, stigma toward AD may vary across age groups and cultural contexts. Future research should aim to include more diverse and representative samples of the populations being studied.

Despite these limitations, the study provides potentially important insights into the psychosocial factors influencing stigma toward individuals with AD and highlights the possibly central role of empathy as a mediator in these relationships, which might help to inform intervention efforts to reduce this stigma.

## Figures and Tables

**Table 1:** Pearson correlation among study variables.

	1	2	3	4	5
AD Stigma	-				
2. Proximity	0.17 ***	-			
3. Empathy	−0.40 ***	−0.06	-		
4. Moral Internalization	−0.27 ***	−0.06	0.62 ***	-	
5. Moral Symbolization	0.02	−0.09	0.29 ***	0.22 ***	-
6. Identity Distress	0.16 **	−0.09	−0.13 **	−0.10 *	0.001

Note: * *p* < 0.05, ** *p* < 0.01, and *** *p* < 0.001.

## Data Availability

The data sets presented in this article are not readily available because participants were informed that data would not be shared beyond the research team, as noted in the consent form.

## Abbreviations

The following abbreviations are used in this manuscript:

AD	Alzheimer’s Disease Dementia
NIA-AA	National Institute on Aging-Alzheimer’s Association

## References

[B1-behavsci-16-00525] Alzheimer’s Association (2024). Alzheimer’s disease facts and figures.

[B2-behavsci-16-00525] Alzheimer’s Disease International (2019). Attitudes to dementia.

[B3-behavsci-16-00525] American Psychiatric Association (2022). Diagnostic and statistical manual of mental disorders *(5th-TR)*.

[B4-behavsci-16-00525] Aquino K., Reed A. (2002). The self-importance of moral identity. Journal of Personality and Social Psychology.

[B5-behavsci-16-00525] Ayalon L., Areán P. A. (2004). Knowledge of Alzheimer’s disease in four ethnic groups of older adults. International Journal of Geriatric Psychiatry.

[B6-behavsci-16-00525] Baron R. M., Kenny D. A. (1986). The moderator–mediator variable distinction in social psychological research: Conceptual, strategic, and statistical considerations. Journal of Personality and Social Psychology.

[B7-behavsci-16-00525] Batson C. D., Lishner D. A., Stocks E. L. (2015). The empathy-altruism hypothesis. The Oxford handbook of prosocial behavior.

[B8-behavsci-16-00525] Batson C. D., O’Quin K., Fultz J., Vanderplas M., Isen A. M. (1983). Influence of self-reported distress and empathy on egoistic versus altruistic motivation to help. Journal of Personality and Social Psychology.

[B9-behavsci-16-00525] Batson C. D., Polycarpou M. P., Harmon-Jones E., Imhoff H. J., Mitchener E. C., Bednar L. L., Klein T. R., Highberger L. (1997). Empathy and attitudes: Can feeling for a member of a stigmatized group improve feelings toward the group?. Journal of Personality and Social Psychology.

[B10-behavsci-16-00525] Berman S. L., Hupp S., Jewell J. (2020). Identity Distress. The encyclopedia of child and adolescent development.

[B11-behavsci-16-00525] Berman S. L., Montgomery M. J., Kurtines W. M. (2004). The development and validation of a measure of identity distress. Identity: An International Journal of Theory and Research.

[B12-behavsci-16-00525] Blay S. L., Peluso É. T. P. (2010). Public stigma: The community’s tolerance of alzheimer disease. The American Journal of Geriatric Psychiatry.

[B13-behavsci-16-00525] Carrera P., Oceja L., Caballero A., Muñoz D., López-Pérez B., Ambrona T. (2012). I feel so sorry! Tapping the joint influence of empathy and personal distress on helping behavior. Motivation and Emotion.

[B14-behavsci-16-00525] Cipriani G., Borin G. (2014). Understanding dementia in the sociocultural context: A review. International Journal of Social Psychiatry.

[B15-behavsci-16-00525] Clinton A. J., Pollini R. A. (2021). Using positive empathy interventions to reduce stigma toward people who inject drugs. Frontiers in Psychology.

[B16-behavsci-16-00525] Cohen M., Werner P., Azaiza F. (2009). Emotional reactions of Arab lay persons to a person with Alzheimer’s disease. Aging & Mental Health.

[B17-behavsci-16-00525] Corner L., Bond J. (2004). Being at risk of dementia: Fears and anxieties of older adults. Journal of Aging Studies.

[B18-behavsci-16-00525] Corrigan P., Markowitz F. E., Watson A., Rowan D., Kubiak M. A. (2003). An attribution model of public discrimination towards persons with mental illness. Journal of Health and Social Behavior.

[B19-behavsci-16-00525] Decety J., Cowell J. M. (2015). Empathy, justice, and moral behavior. AJOB Neuroscience.

[B20-behavsci-16-00525] Decety J., Jackson P. L. (2004). The functional architecture of human empathy. Behavioral and Cognitive Neuroscience Reviews.

[B21-behavsci-16-00525] Ding W., Shao Y., Sun B., Xie R., Li W., Wang X. (2018). How can prosocial behavior be motivated? The different roles of moral judgment, moral elevation, and moral identity among the young Chinese. Frontiers in Psychology.

[B22-behavsci-16-00525] Dzumba D., Cantu C., Humphries N., Kovacks A., Shelly C., Bailey T., Barnett M. (2018). Moral identity internalization, not symbolization, is associated with lower ageism among younger adults. Innovation in Aging.

[B23-behavsci-16-00525] Goffman E. (1963). Stigma; notes on the management of spoiled identity.

[B24-behavsci-16-00525] Gotowiec S., van Mastrigt S. (2019). Having versus doing: The roles of moral identity internalization and symbolization for prosocial behaviors. The Journal of Social Psychology.

[B25-behavsci-16-00525] Hamed W. E., Babegi A. S., Elamin H. A. M., Kamel N. A., Escolano-Castillo A. M., Dailah H. G., Eletreby R. R. (2025). Stigmatizing attitudes and predictors of empathy toward mentally ill patients among psychiatric and mental health nurses. BMC Nursing.

[B26-behavsci-16-00525] Harper L., Dobbs B. M., Stites S. D., Sajatovic M., Buckwalter K. C., Burgener S. C. (2019). Stigma in dementia: It’s time to talk about it. Current Psychiatry.

[B27-behavsci-16-00525] Hassan E., Hicks B., Tabet N., Farina N. (2024). Factors associated with dementia-related stigma in British adolescents. BMC Public Health.

[B28-behavsci-16-00525] Ito K., Tsuda S. (2025). Effects of clinical stage, behavioral and psychological symptoms of dementia, and living arrangement on social distance towards people with dementia. PLoS ONE.

[B29-behavsci-16-00525] Jack C. R., Bennett D. A., Blennow K., Carrillo M. C., Dunn B., Haeberlein S. B., Holtzman D. M., Jagust W., Jessen F., Karlawish J., Liu E., Molinuevo J. L., Montine T., Phelps C., Rankin K. P., Rowe C. C., Scheltens P., Siemers E., Snyder H. M., Sperling R. (2018). NIA-AA research framework: Toward a biological definition of alzheimer’s disease. Alzheimer’s & Dementia: The Journal of the Alzheimer’s Association.

[B30-behavsci-16-00525] Johnson R., Harkins K., Cary M., Sankar P., Karlawish J. (2015). The relative contributions of disease label and disease prognosis to Alzheimer’s stigma: A vignette-based experiment. Social Science & Medicine.

[B31-behavsci-16-00525] Kleinman A., Hall-Clifford R. (2009). Stigma: A social, cultural and moral process. Journal of Epidemiology and Community Health.

[B32-behavsci-16-00525] Low L.-F., Purwaningrum F. (2020). Negative stereotypes, fear and social distance: A systematic review of depictions of dementia in popular culture in the context of stigma. BMC Geriatrics.

[B33-behavsci-16-00525] MacRae H. (1999). Managing courtesy stigma: The case of alzheimer’s disease. Sociology of Health & Illness.

[B34-behavsci-16-00525] Nguyen T., Li X. (2020). Understanding public-stigma and self-stigma in the context of dementia: A systematic review of the global literature. Dementia.

[B35-behavsci-16-00525] O’Connor M. L., McFadden S. H. (2012). A terror management perspective on young adults’ ageism and attitudes toward dementia. Educational Gerontology.

[B36-behavsci-16-00525] Oscar N., Fox P. A., Croucher R., Wernick R., Keune J., Hooker K. (2017). Machine learning, sentiment analysis, and tweets: An examination of alzheimer’s disease stigma on twitter. The Journals of Gerontology: Series B.

[B37-behavsci-16-00525] Peng L., Jiang Y., Ye J., Xiong Z. (2024). The impact of empathy on prosocial behavior among college students: The mediating role of moral identity and the moderating role of sense of security. Behavioral Sciences.

[B38-behavsci-16-00525] Piver L. C., Nubukpo P., Faure A., Dumoitier N., Couratier P., Clément J.-P. (2012). Describing perceived stigma against Alzheimer’s disease in a general population in France: The STIG-MA survey. International Journal of Geriatric Psychiatry.

[B39-behavsci-16-00525] Pryor J. B., Reeder G. D., Monroe A. E., Patel A. (2009). Stigmas and prosocial behavior. The psychology of prosocial behavior: Group processes, intergroup relations, and helping.

[B40-behavsci-16-00525] Quinn D. M., Chaudoir S. R. (2009). Living with a concealable stigmatized identity: The impact of anticipated stigma, centrality, salience, and cultural stigma on psychological distress and health. Journal of Personality and Social Psychology.

[B41-behavsci-16-00525] Rewerska-Juśko M., Rejdak K. (2020). Social stigma of people with dementia. Journal of Alzheimer’s Disease.

[B42-behavsci-16-00525] Reynolds S. J., Ceranic T. L. (2007). The effects of moral judgment and moral identity on moral behavior: An empirical examination of the moral individual. The Journal of Applied Psychology.

[B43-behavsci-16-00525] Rosin E. R., Blasco D., Pilozzi A. R., Yang L. H., Huang X. (2020). A narrative review of alzheimer’s disease stigma. Journal of Alzheimer’s Disease.

[B44-behavsci-16-00525] Spreng R. N., McKinnon M. C., Mar R. A., Levine B. (2009). The toronto empathy questionnaire: Scale development and initial validation of a factor-analytic solution to multiple empathy measures. Journal of Personality Assessment.

[B45-behavsci-16-00525] Stites S. D., Largent E. A., Johnson R., Harkins K., Karlawish J. (2021). Effects of self-identification as a caregiver on expectations of public stigma of alzheimer’s disease. Journal of Alzheimer’s Disease Reports.

[B46-behavsci-16-00525] Stites S. D., Rubright J. D., Karlawish J. (2018). What features of stigma do the public most commonly attribute to Alzheimer’s disease dementia? Results of a survey of the U.S. general public. Alzheimer’s & Dementia.

[B47-behavsci-16-00525] Teichmann B., Gkioka M., Kruse A., Tsolaki M. (2022). Informal caregivers’ attitude toward dementia: The impact of dementia knowledge, confidence in dementia care, and the behavioral and psychological symptoms of the person with dementia. A cross-sectional study. Journal of Alzheimer’s Disease: JAD.

[B48-behavsci-16-00525] Urbańska K., Szcześniak D., Rymaszewska J. (2015). The stigma of dementia. Postępy Psychiatrii I Neurologii.

[B49-behavsci-16-00525] Weiner M. F., Lipton A. M., American Psychiatric Publishing (2009). The American Psychiatric Publishing textbook of Alzheimer disease and other dementias.

[B50-behavsci-16-00525] Werner P. (2005). Social distance towards a person with Alzheimer’s disease. International Journal of Geriatric Psychiatry.

[B51-behavsci-16-00525] Werner P., Davidson M. (2004). Emotional reactions of lay persons to someone with Alzheimer’s disease. International Journal of Geriatric Psychiatry.

[B52-behavsci-16-00525] Werner P., Giveon S. M. (2008). Discriminatory behavior of family physicians toward a person with Alzheimer’s disease. International Psychogeriatrics.

[B53-behavsci-16-00525] Winterich K. P., Aquino K., Mittal V., Swartz R. (2013). When moral identity symbolization motivates prosocial behavior: The role of recognition and moral identity internalization. Journal of Applied Psychology.

[B54-behavsci-16-00525] Yang L. H., Kleinman A., Link B. G., Phelan J. C., Lee S., Good B. (2007). Culture and stigma: Adding moral experience to stigma theory. Social Science & Medicine.

